# Minimal Absent Words in Prokaryotic and Eukaryotic Genomes

**DOI:** 10.1371/journal.pone.0016065

**Published:** 2011-01-31

**Authors:** Sara P. Garcia, Armando J. Pinho, João M. O. S. Rodrigues, Carlos A. C. Bastos, Paulo J. S. G. Ferreira

**Affiliations:** 1 Signal Processing Laboratory, IEETA, University of Aveiro, Aveiro, Portugal; 2 Department of Electronics, Telecommunications and Informatics, University of Aveiro, Aveiro, Portugal; Kyushu Institute of Technology, Japan

## Abstract

Minimal absent words have been computed in genomes of organisms from all domains of life. Here, we explore different sets of minimal absent words in the genomes of 22 organisms (one archaeota, thirteen bacteria and eight eukaryotes). We investigate if the mutational biases that may explain the deficit of the shortest absent words in vertebrates are also pervasive in other absent words, namely in minimal absent words, as well as to other organisms. We find that the compositional biases observed for the shortest absent words in vertebrates are not uniform throughout different sets of minimal absent words. We further investigate the hypothesis of the inheritance of minimal absent words through common ancestry from the similarity in dinucleotide relative abundances of different sets of minimal absent words, and find that this inheritance may be exclusive to vertebrates.

## Introduction

The set of absent words of a sequence is the set of all words that cannot be found in the sequence. This set is too large and of limited interest for practical purposes. Hence, we have introduced the concept of *minimal absent words* that have the following property: the new word formed by removing the left- or rightmost character from a minimal absent word is no longer an absent word [1].

Minimal absent words are defined to have at least 3 characters and have been computed in genomes of organisms from all domains of life. The core of a minimal absent word, i.e. the word that remains if its left- and rightmost characters are removed, is a maximal repeat. A maximal repeat is a perfect repeat (without gaps or misspellings) that occurs at least twice and which cannot be further extended to either its left- or right-end side without loss of similarity.

Minimal absent words are a generalization of the short absent words introduced by Hampikian and Andersen under the term *nullomers* [2], and by Herold *et al.* as *unwords* [3]. For sequences with all letters and pairs of letters of the alphabet, the set of nullomers/unwords will correspond to the shortest minimal absent words.

For illustration, consider the sequence GCTAACCGATG and its reverse complement. The set of minimal absent words of this sequence concatenated with its reverse complement (with artificial words across the boundary ignored) is {AAA, AAG, AAT, ACA, ACG, ACT, AGA, AGG, AGT, ATA, ATT, CAA, CAC, CAG, CCA, CCC, CCT, CGC, CGT, CTC, CTG, CTT, GAA, GAC, GAG, GCA, GCC, GCG, GGA, GGC, GGG, GTA, GTC, GTG, TAC, TAT, TCA, TCC, TCT, TGA, TGC, TGG, TGT, TTC, TTG, TTT, AGCT, CATG, CCGG, CTAG, GATC, TCGA, TTAA}, whereas its set of nullomers/unwords solely includes the trinucleotides of the above set. Moreover, the set of maximal repeats in this sequence concatenated with its reverse complement is {A, C, G, T, AT, CG, GC, TA}.

The minimality constraint imposed on minimal absent words guarantees that amongst all absent words, minimal absent ones are the closest to the boundary of the set of all occurring words.

An important question concerning absent words is their biological relevance. Speculation of negative selection acting upon nullomers led Hampikian and Andersen to envisage a range of potential applications [2]. Herold *et al.* suggested that unwords may not have a functional meaning but might be useful for large scale mutagenesis experiments [3]. We previously hypothesized that minimal absent words might be used as biomarkers at the individual level, or for the comparison of genetic traits at the species or population level [1]. However, the most comprehensive analysis so far, to the best of our knowledge, on the biological implications of absent words is authored by Acquisti *et al.*, who carefully analyzed the set of nullomers/unwords of length 11 base pairs (bp) in the human genome, and questioned the evidence for assuming those words to be under negative selective pressure [4]. Instead, they proposed that the mutational characteristics of the genome, namely the hypermutability (hence deficit) of CpGs in vertebrates, provides a reasonable explanation for the multiple CpGs observed in all of the shortest absent words in the human and other mammalian genomes [4]. Moreover, Acquisti *et al.* hypothesized that regular point mutations, in addition to hypermutable CpGs, are an important justification for the presence of nullomers/unwords [4]. They compared the list of nullomers/unwords in the human and other mammalian genomes and found that the human genome shares more nullomers/unwords with its closest evolutionary relative, the chimpanzee, than with more distantly related mammals, hence suggesting that the set of human nullomers/unwords contains nullomers/unwords inherited from the common ancestor of human and chimpanzee, in addition to those that have arisen within the human lineage [4].

Here, we complement their analysis by investigating if the compositional biases that may explain the deficit of the shortest absent words in vertebrates are also pervasive in other absent words, namely, in minimal absent words. Moreover, we compare sets of minimal absent words, and respective compositional biases, in organisms other than vertebrates. We further investigate the hypothesis of the inheritance of minimal absent words through common ancestry, in addition to lineage specific inheritance, from the similarity in dinucleotide compositional biases of different sets of minimal absent words. For estimating the compositional biases, we use the methodology of dinucleotide relative abundances pioneered by Brendel *et al.* [5], Pietrokovski *et al.* [6], and Karlin and collaborators (e.g. [7]–[9]).

## Methods

### Genomic data

We considered the genomes of one archaeota, thirteen bacteria and eight case-study eukaryotes (Table 1) as available from the NCBI database [10], the *Saccharomyces* Genome Database [11], the database in The Arabidopsis Information Resource [12], the WormBase database [13], and the FlyBase database [14]. For convenience, the scientific names in figures and tables are abbreviated to the first letter of the genus followed by the first letter of the epithet. Two exceptions include two additional letters as prefixes, namely for the methicillin-resistant *Staphylococcus aureus* (MRSa) and the methicillin-susceptible *Staphylococcus aureus* (MSSa). The reference assemblies of the reported NCBI builds are used for the chicken, mouse, chimpanzee and human genomes.

**Table 1:** Genomic data.

Organism	Abbreviation	Genome reference
**Euryarchaeota**		
*Methanococcus jannaschii* strain DSM2661	Mj	NC000909
**Bacteria**		
*Bacillus anthracis* strain Ames	Ba	NC003997
*Bacillus subtilis* strain 168	Bs	NC000964
*Escherichia coli* strain K-12 substrain MG1655	Ec	NC000913
*Haemophilus influenzae* strain Rd KW20	Hi	NC000907
*Helicobacter pylori* strain 26695	Hp	NC000915
*Lactobacillus casei* strain BL23	Lc	NC010999
*Lactococcus lactis* strain Il1403	Ll	NC002662
*Mycoplasma genitalium* strain G37	Mg	NC000908
*Staphylococcus aureus* strain N315	Sa	NC002745
methicillin-resistant *Staphylococcus aureus* strain 252	MRSa	NC002952
methicillin-susceptible *Staphylococcus aureus* strain 476	MSSa	NC002953
*Streptococcus pneumoniae* strain CGSP14	Sp	NC010582
*Xanthomonas campestris* strain 8004	Xc	NC007086
**Eukaryotes**		
*Saccharomyces cerevisiae* strain S228C (budding yeast)	Sc	SGD release 1
*Arabidopsis thaliana* (thale cress)	At	AGI release 7.2
*Caenorhabditis elegans* (worm)	Ce	WormBase release 170
*Drosophila melanogaster* (fruit fly)	Dm	FlyBase release 5
*Gallus gallus* (chicken)	Gg	build 2.1
*Mus musculus* (mouse)	Mm	build 37.1
*Pan troglodytes* (chimpanzee)	Pt	build 2.1
*Homo sapiens* (human)	Hs	build 36.3

Organisms selected for this study, with reference to the respective abbreviation, and identification of genome sequence data by accession number (euryarchaeota and bacteria) or genome assembly project (eukaryotes).

### Finding minimal absent words

Consider a finite alphabet 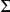 with cardinality 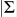. Let 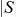 denote the length of a string 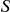 over 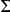 and 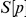 its 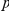 th character, with 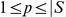. A substring of 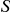 starting at position 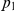 and ending at position 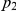 is denoted by 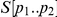, with 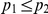. If 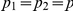, then 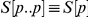. Moreover, 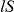 (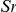) denotes the concatenation of character 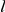 (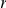) to the left (right) end side of 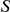, with 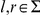.

Let 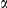 denote a substring of 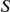 and 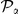 denote the set of positions of 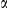 in 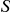, i.e. 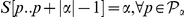 and 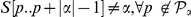. A maximal repeated pair in 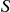 is a pair of identical substrings such that the character to the immediate left (right) of one of the substrings is different from the character to the immediate left (right) of the other substring, i.e. a triple 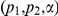, such that 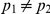, 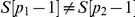 and 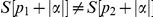, with 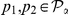 [15]. A substring 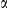 is a maximal repeat in 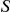 if it occurs in a maximal pair, i.e. if there is at least a maximal repeated pair in 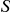 of the form (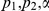), with 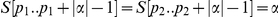 [15].

A string 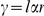 is a minimal absent word of 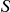 if and only if 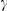 is not a substring of 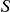, but 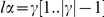 and 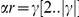 are substrings of 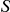. For convenience, we consider 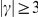.

#### Theorem 1

(proof in [1]) *If 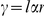 is a minimal absent word of 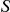, then 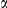 is a maximal repeat in 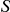
*.

#### Theorem 2

(proof in [1]) *A string 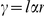 is a minimal absent word of 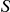 if and only if 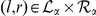 but 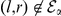, where 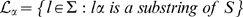, 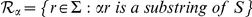 and 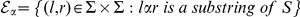
*.

If 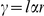 is a minimal absent word of 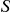, then 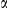 occurs at least twice in 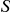 and these occurrences may partially overlap. It is easily verifiable that, as 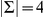 in DNA sequences, the maximum number of minimal absent words associated to a particular 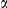 is twelve, and it occurs when 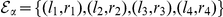, with 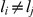 and 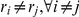. This property implies that frequent repeats have a high probability of not generating minimal absent words, because for those frequent repeats 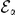 is often equal to 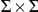.

Minimal absent words are found by reading the information in a suffix array. A suffix array is an array of integers 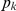, with 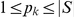 and 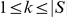, each pointing to the beginning of a suffix of 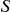, such that 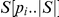 lexicographically precedes 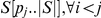. Two auxiliary arrays are used, namely, the longest common prefix (lcp) array, and the left character (bwt) array, the latter corresponding to the Burrows and Wheeler transform [16]. The lcp-array contains the lengths of the longest common prefix between consecutive ordered suffixes, i.e. 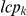 indicates the length of the longest common prefix between 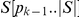 and 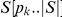, with 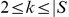. By convention, 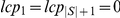. The bwt-array is a permutation of 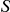 such that 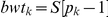 if 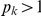, and, by convention, 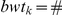 if 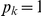, where 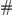 is a character that does not belong to the alphabet 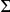. Conceptually, the bwt-array does not provide any additional information, as the left character of any character of 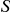 can be determined by direct access to 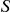. However, the bwt-array allows for sequential memory access, hence improving the performance due to enhanced use of cache [17].

The first part of the algorithm generates all lcp-intervals using the lcp-array and a stack, and is adapted from [18] and [17]. An lcp-interval of lcp-depth 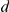 is the interval 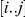, with 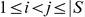, if and only if 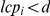; 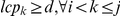; 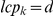, for at least one 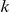 in 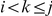; and 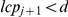. Each lcp-interval delimits a subset of suffixes that start with a common 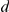-letter prefix 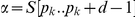, 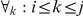. The second part of the algorithm determines if an lcp-interval is left-diverse, i.e. if at least two characters of 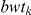 differ, for 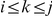. In that case, 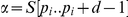 is a maximal repeat, as all substrings 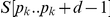 are identical, 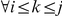. From these maximal repeats, all minimal absent words associated to each lcp-interval are computed and then output. See [1] for details on the algorithm.

Sets of minimal absent words are found by concatenating the genome with its reverse complement using a delimiting character that does not belong to the alphabet, to avoid the formation of artificial words across boundaries. The order by which the chromosomes are inserted is irrelevant. We solely consider unambiguous nucleotides (A, C, G or T) and have ignored all sequence ambiguities by replacing every subsequence of ambiguously sequenced nucleotides (e.g. K, M, N, R, S, W and Y) with a delimiting character that does not belong to the alphabet.

### Compositional biases from dinucleotide relative abundances

Let 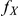 denote the relative frequency of nucleotide 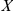 in a given genomic sequence, and 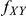 the relative frequency of dinucleotide 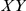. A standard assessment of nucleotide bias is through the odds-ratio
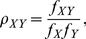
(1)with 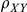 values sufficiently larger (smaller) than one implying that the 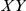 dinucleotide is considered of high (low) relative abundance compared to a random association of its component mononucleotides [19].

For double-stranded DNA molecules, (1) must be modified in order to account for the inherent complementary anti-parallel structure. Let 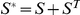 define the string resulting from combining the DNA sequence 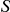 with its reverse complement 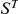. In 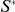, the analogous strand symmetric functionals for the base frequencies are now
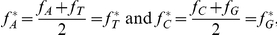
with 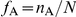, where 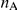 is the number of adenine (A) nucleotides in a sequence of length 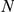, with equivalent formulas for cytosine (C), guanine (G), and thymine (T). The analogous strand symmetric functionals for the dinucleotide odds-ratio are now
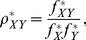
(2)an example being
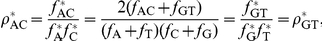
with
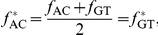
and 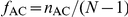, where 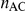 is the number of AC dinucleotides in a sequence of length 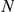, with equivalent formulas for all other dinucleotides. The total number of dinucleotides in a set with cardinality 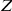 of minimal absent words of word length 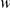 is 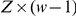. The vector of 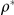 values has remarkably low variance throughout the genome of a given organism, and can discriminate sequences from distinct organisms [20]. Dinucleotide relative abundances are estimated considering overlapping, i.e. word ACTAC may be segmented into four dinucleotides, namely two dinucleotides AC, one dinucleotide CT, and one dinucleotide TA.

## Results and Discussion

### The total number of minimal absent words increases with genome size

**Figure 1:**
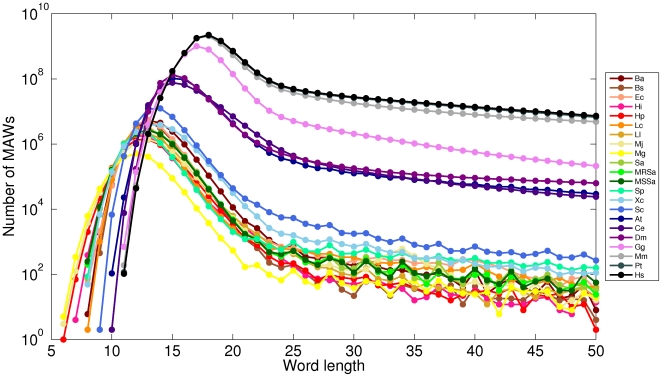
Number of minimal absent words in genomic sequences:. Distributions of the number of minimal absent words (MAWs) in the genomes of selected organisms for word length up to 50.

Figure 1 displays the distributions of the number of minimal absent words in the genomes of selected organisms for increasing word length. We sampled the distributions at word length 11 (the resulting set of minimal absent words being designated 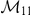), which roughly coincides with the beginning of the curves and allows for the comparison with previous studies [4]; at word length 14 (the resulting set of minimal absent words being designated 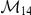), as it is close to the peak of the distribution for most prokaryotic genomes surveyed; at word length 17 (the resulting set of minimal absent words being designated 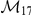), as it is close to the peak of the distribution for most genomes of higher eukaryotes surveyed; and at word length 24 (the resulting set of minimal absent words being designated 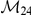) for sampling the distributions at the beginning of the right-end tails. These right-end tails are the main differences to profiles obtained for artificially generated DNA strings with a random distribution of the four unambiguous nucleotides (A, C, G and T) [1].

### Compositional biases are not uniform throughout different sets of minimal absent words

Table 2 reports the GC content (denoted by G+C) of the genome and respective sets of minimal absent words in each organism, with 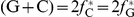 and 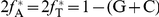. As a consequence of ignoring sequence ambiguities, the final (haploid) genome size in units of base pairs (bp) may differ slightly from values commonly reported in the literature. We also report the cardinality (size) of each set of minimal absent words, i.e. the total number of minimal absent words in the set. Table 3 displays the dinucleotide relative abundances of the sets of minimal absent words in the genome of each organism. The reported values are the strand symmetric functionals, with 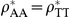 denoted by AA and TT, and so on. The counts were estimated for each word separately, and cumulative values were estimated over the entire set.

**Table 2:** Compositional biases.

	Genome		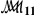		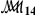		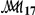		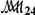	
	Size (bp)	G+C	Size	G+C	Size	G+C	Size	G+C	Size	G+C
Ba	5,227,293	0.36	873,180	0.56	4,485,825	0.32	330,770	0.26	694	0.36
Bs	4,214,630	0.44	935,554	0.54	3,089,102	0.40	126,496	0.36	162	0.40
Ec	4,639,675	0.50	839,014	0.50	3,504,611	0.52	141,556	0.54	702	0.54
Hi	1,830,023	0.38	888,594	0.48	947,347	0.32	43,098	0.32	312	0.38
Hp	1,667,825	0.38	722,762	0.48	943,139	0.36	71,452	0.32	218	0.36
Lc	3,079,196	0.46	1,048,894	0.52	1,715,556	0.44	49,896	0.44	630	0.54
Ll	2,365,589	0.36	919,156	0.50	1,446,921	0.30	93,684	0.26	516	0.28
Mj	1,664,957	0.32	539,920	0.46	1,051,171	0.28	96,626	0.22	428	0.32
Mg	580,076	0.32	391,682	0.40	215,544	0.26	10,658	0.24	66	0.42
Sa	2,814,816	0.32	852,402	0.50	1,969,819	0.28	123,642	0.24	362	0.34
MRSa	2,902,619	0.32	852,542	0.50	1,988,247	0.28	131,756	0.24	354	0.34
MSSa	2,799,802	0.32	851,978	0.50	1,988,247	0.28	126,348	0.22	418	0.36
Sp	2,209,198	0.40	1,019,764	0.50	1,078,877	0.36	39,170	0.32	576	0.38
Xc	5,148,708	0.64	853,780	0.48	3,936,720	0.66	642,460	0.72	1,762	0.66
Sc	12,739,648	0.38	423,500	0.64	12,398,033	0.38	901,182	0.28	5,619	0.20
At	118,973,747	0.36	22,900	0.80	56,034,743	0.48	56,311,864	0.30	363,823	0.28
Ce	100,269,917	0.36	7,668	0.70	53,359,766	0.48	43,423,752	0.30	648,884	0.28
Dm	162,348,295	0.42	104	0.70	74,357,742	0.50	54,260,892	0.36	506,678	0.34
Gg	984,856,238	0.42	700	0.60	38,646,642	0.56	1,006,332,266	0.42	6,768,820	0.36
Mm	2,559,165,832	0.42	190	0.62	26,244,051	0.56	1,788,521,026	0.44	47,781,970	0.40
Pt	2,752,354,403	0.40	116	0.62	26,194,501	0.56	1,767,172,092	0.44	58,934,573	0.36
Hs	2,858,029,377	0.40	104	0.60	25,778,756	0.56	1,788,484,146	0.44	61,816,985	0.36

GC content (G+C) and total (haploid) genome size in units of base pairs (bp) for selected genomes, followed by the GC content and total number of words (size) in sets of minimal absent words of word length 11 (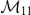), 14 (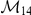), 17 (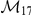) and 24 (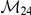).

**Table 3:** Dinucleotide relative abundances.

	TT	GT	CT		TG	GG		TC			TT	GT	CT		TG	GG		TC		
	AA	AC	AG	AT	CA	CC	CG	GA	GC	TA	AA	AC	AG	AT	CA	CC	CG	GA	GC	TA
	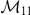										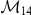									
Ba	0.8674	1.0829	1.0923	0.9579	1.0367	0.9270	0.9426	1.0532	0.9268	1.0778	1.1632	0.8850	0.9131	0.9778	1.0685	0.9252	0.9343	0.9982	1.1398	0.8450
Bs	0.8389	1.1625	1.0671	0.9096	0.9544	0.9941	0.9771	0.9754	0.8734	1.2592	1.3249	0.6594	0.8978	1.0097	1.1116	0.9227	1.0001	1.0920	1.3769	0.5639
Ec	0.8653	1.0761	1.1074	0.9460	0.9447	1.0551	0.8959	1.0539	0.8099	1.1434	1.2753	0.8326	0.7758	1.1317	1.1428	0.8806	1.2016	0.8909	1.3901	0.6799
Hi	0.9717	1.0392	1.0115	1.0223	1.0561	0.8915	0.9939	0.9865	1.0328	1.0298	1.3343	0.7593	0.7542	0.9443	1.1501	1.0040	1.0393	0.8656	1.5976	0.6944
Hp	0.9999	0.8946	1.0342	1.1011	1.0897	0.9942	0.8301	0.9418	1.1336	1.0129	1.4790	0.5966	0.9617	0.8030	0.9171	1.1756	0.9360	0.8583	1.6716	0.6833
Lc	0.8694	1.0764	1.0688	0.9881	0.9256	0.9919	1.0015	1.0188	0.9064	1.2046	1.3264	0.7931	0.7564	1.0573	1.2951	0.8888	1.0202	0.9474	1.4023	0.4950
Ll	0.9394	1.0483	1.0543	1.0123	1.0562	0.9297	0.9069	1.0110	0.9543	1.0446	1.3057	0.7430	0.9376	0.8808	1.1690	0.9871	0.5607	1.0806	1.1910	0.6240
Mj	1.0080	0.9651	1.0950	1.0086	1.1583	1.0592	0.5254	0.9743	0.9255	0.9509	1.1675	0.6589	1.1160	0.9607	0.9928	1.3265	0.1362	1.0894	1.0554	0.8397
Mg	1.0729	0.9656	1.0820	0.9640	1.1730	1.0249	0.4096	0.9425	0.9614	0.9203	1.3516	0.9641	1.0373	0.6887	1.1480	0.9439	0.2021	0.8445	1.2541	0.6935
Sa	0.9518	1.0724	1.0640	0.9759	1.0598	0.8798	0.9294	1.0273	0.9538	1.0301	1.1225	0.9195	0.8297	1.0227	1.2305	0.8174	0.7703	0.9571	1.2221	0.8439
MRSa	0.9512	1.0725	1.0643	0.9753	1.0583	0.8838	0.9293	1.0283	0.9506	1.0308	1.1228	0.9182	0.8331	1.0222	1.2262	0.8181	0.7731	0.9591	1.2198	0.8442
MSSa	0.9510	1.0714	1.0641	0.9781	1.0582	0.8792	0.9316	1.0292	0.9530	1.0308	1.1229	0.9187	0.8311	1.0221	1.2291	0.8185	0.7690	0.9570	1.2240	0.8440
Sp	0.9238	1.0612	1.0455	1.0114	1.0453	0.9319	0.9319	1.0047	0.9616	1.0690	1.2614	0.7412	1.0843	0.8686	1.1094	1.0437	0.4806	1.1595	1.0175	0.6272
Xc	0.9980	1.0233	1.0372	0.9046	0.9933	1.0837	0.9363	1.0844	0.8420	0.8905	0.9987	0.9314	0.8578	1.2561	1.3101	0.7968	1.1579	0.9357	1.3168	0.3718
Sc	0.7361	1.1548	1.0227	0.9554	0.8656	0.9264	1.1417	1.0688	0.9380	1.3886	1.1430	0.8883	0.9940	0.9657	1.1245	1.0058	0.6842	1.0659	0.9637	0.7815
At	0.6870	1.1120	0.9022	1.0019	0.8643	0.9710	1.1041	0.7442	1.0904	2.4828	0.9740	1.0247	1.0508	0.9970	1.0531	0.9553	0.8830	1.0528	0.9136	0.9670
Ce	0.7467	1.0779	1.1695	0.7336	0.5345	1.0924	1.0418	0.6806	0.9795	3.1638	0.9972	1.0064	1.0168	1.0173	1.0793	0.9359	0.9132	1.0663	0.9413	0.9046
Dm	0.2031	1.4083	1.3361	0.3250	0.1444	1.1315	1.1395	0.5597	0.8185	4.6312	0.9694	1.0001	1.0178	1.0264	1.0255	0.9717	0.9731	1.0342	0.9837	0.9804
Gg	0.7215	1.2696	0.4839	1.3320	0.1294	0.3009	2.7760	1.6576	1.0173	1.7020	0.9875	1.0633	0.8567	1.1065	0.8177	0.9293	1.3506	1.0541	0.9569	1.1795
Mm	0.8047	1.4168	0.0980	1.6093	0.0802	0.1989	3.1720	1.4792	1.1052	2.0116	1.0112	1.1047	0.7685	1.1408	0.7452	0.8347	1.5629	1.0451	1.0283	1.2707
Pt	0.5187	1.4746	0.2044	1.7917	0.0292	0.2080	3.1465	1.7082	0.8499	1.9804	0.9765	1.1250	0.7935	1.1155	0.7496	0.8127	1.5620	1.0659	1.0202	1.2721
Hs	0.6270	1.5013	0.1453	1.5549	0.0969	0.2078	3.1589	1.4529	0.9975	2.3073	0.9770	1.1258	0.7904	1.1168	0.7466	0.8113	1.5697	1.0670	1.0197	1.2732
	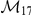										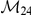									
Ba	1.2532	0.7720	0.9573	0.8747	1.0238	0.9385	0.8698	1.0798	1.2244	0.7420	1.2009	0.8422	0.9177	0.9518	0.8565	1.0275	1.3330	1.0763	1.0910	0.8396
Bs	1.4571	0.5755	0.9314	0.8589	1.0798	0.9053	0.9921	1.1213	1.5339	0.4535	1.2538	0.7191	1.0631	0.8939	1.0631	0.9644	0.8647	1.0943	1.2904	0.6419
Ec	1.3243	0.7623	0.8105	1.1538	1.1810	0.8463	1.1719	0.8565	1.4930	0.6207	1.0957	0.8330	0.8121	1.2909	1.0156	0.8584	1.3054	0.8330	1.4561	1.0463
Hi	1.3925	0.7487	0.7372	0.8735	1.1157	1.0156	1.1798	0.8351	1.7471	0.6578	1.6130	0.8853	0.7172	0.6313	1.0249	1.2034	1.1918	0.6367	1.5788	0.6023
Hp	1.6071	0.5276	1.0325	0.6202	0.7679	1.2301	1.0894	0.8674	1.9277	0.5925	1.4888	0.6517	1.0665	0.6898	0.8469	1.0904	1.0143	0.9968	1.4454	0.6208
Lc	1.3740	0.7606	0.7478	1.0331	1.3132	0.8841	1.0198	0.9273	1.4838	0.4472	1.3225	0.9470	1.0247	0.6921	0.9837	0.9648	1.0462	0.8282	1.2417	0.8722
Ll	1.3628	0.6761	0.9463	0.7994	1.1622	0.9457	0.4694	1.1463	1.2745	0.5643	1.2463	1.1724	1.2465	0.6195	1.2349	0.3842	0.2861	1.1048	0.8257	0.6208
Mj	1.2579	0.5432	1.1750	0.8504	0.8545	1.4438	0.1240	1.1772	1.1479	0.7630	1.3002	0.6199	0.9459	0.9135	0.7801	1.6623	0.9276	1.0307	0.9851	0.7976
Mg	1.3999	0.9789	1.0396	0.6213	1.1343	0.9374	0.1796	0.8249	1.3549	0.6482	1.2241	1.1004	0.9062	0.7949	1.1327	1.3469	0.5563	0.7228	0.8492	0.9062
Sa	1.1715	0.8083	0.8213	0.9710	1.2210	0.8432	0.6931	1.0024	1.4168	0.7926	1.1583	0.8588	0.8889	0.9751	1.2354	1.2402	0.4757	0.9836	1.0193	0.7482
MRSa	1.1749	0.8022	0.8264	0.9672	1.2122	0.8459	0.6956	1.0064	1.4191	0.7918	1.2262	0.8094	0.9032	0.9319	1.2391	1.2731	0.4355	1.0450	0.9464	0.6387
MSSa	1.1734	0.8047	0.8238	0.9691	1.2136	0.8474	0.6976	1.0086	1.4048	0.7913	1.1488	0.7910	0.8930	1.0286	1.2138	1.3481	0.4349	0.9586	1.0972	0.7625
Sp	1.3139	0.6674	1.0855	0.8313	1.0782	1.0787	0.4590	1.2045	1.0577	0.5800	1.1551	0.9672	1.1612	0.7942	1.0862	0.7218	0.8312	1.1224	1.0762	0.7239
Xc	0.8915	0.8372	0.9190	1.5356	1.4737	0.7039	1.1750	0.7927	1.4666	0.2290	1.1805	0.7672	0.8686	1.4490	1.0932	0.8619	1.1768	1.0129	1.2730	0.5404
Sc	1.2873	0.7379	0.9707	0.8648	1.0771	1.0636	0.6238	1.1633	0.9710	0.6474	1.1300	0.7822	0.7601	1.0212	1.0169	1.5590	0.8439	0.8460	1.4507	0.9346
At	1.1882	0.8758	1.0276	0.8891	1.1467	0.9497	0.4811	1.1619	0.7885	0.7090	1.3495	0.7949	1.0314	0.7427	0.9481	1.2108	0.7179	1.1111	0.8849	0.6470
Ce	1.3544	0.8051	0.8595	0.8300	1.1331	0.9660	0.8434	1.1434	0.9442	0.5638	1.6479	0.6363	0.6449	0.6548	0.9278	1.4986	1.4449	0.9360	1.4713	0.4245
Dm	1.3113	0.7948	0.8135	0.9479	1.1792	1.0690	0.8591	0.8354	1.4758	0.7088	1.2507	1.0053	0.7587	0.8966	1.2381	1.0439	0.8886	0.7289	1.3756	0.7875
Gg	1.0517	0.8999	1.2162	0.8893	1.2984	1.0510	0.1728	0.9849	1.0822	0.7658	1.4832	0.7527	1.0513	0.6496	1.1000	1.5054	0.1512	0.9242	1.0100	0.5236
Mm	0.9765	0.9321	1.2167	0.9296	1.2398	1.1517	0.2074	0.9810	0.9470	0.8716	1.2105	0.8866	1.1253	0.7965	1.2101	1.3217	0.1254	0.9683	0.8747	0.6859
Pt	0.9868	0.9224	1.1997	0.9418	1.2328	1.1651	0.2274	0.9730	0.9508	0.8739	1.2579	0.8113	1.0631	0.8358	1.1596	1.3135	0.2443	0.9229	1.0897	0.7115
Hs	0.9852	0.9238	1.1992	0.9420	1.2319	1.1646	0.2330	0.9715	0.9515	0.8765	1.2583	0.8108	1.0642	0.8353	1.1585	1.3157	0.2448	0.9251	1.0834	0.7101

Dinucleotide relative abundances for sets of minimal absent words of word length 11 (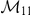), 14 (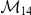), 17 (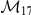) and 24 (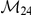).

The compositional biases displayed in Tables 2 and 3 provide additional information for investigating the hypothesis of the hypermutability of CpGs explaining the absence of nullomers/unwords in vertebrate genomes, as proposed by Acquisti *et al.* [4]. We find that this hypothesis needs revision for longer absent words, as neither the base nor dinucleotide compositional biases are uniform throughout sets of minimal absent words of increasing word length. For example, the dinucleotide CG is over-represented in sets 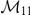 and 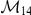 for the vertebrate genomes considered, but under-represented in sets 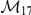 and 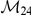. For quantifying the under- or over-representation of a dinucleotide in a given genome, we use the boundaries proposed by Karlin and collaborators, who proved that a conservative estimate of 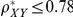 or 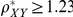, respectively, occurs for sufficiently long (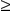 5kb) random sequences, with probability approximately 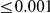, and independent of genome base composition. The rationale follows that, for a random sequence, the 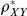 values for all 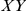 approach one, with deviations of about 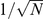 for sequences of length 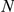 [21].

### The inheritance of minimal absent words through common ancestry may be exclusive to vertebrates

**Figure 2:**
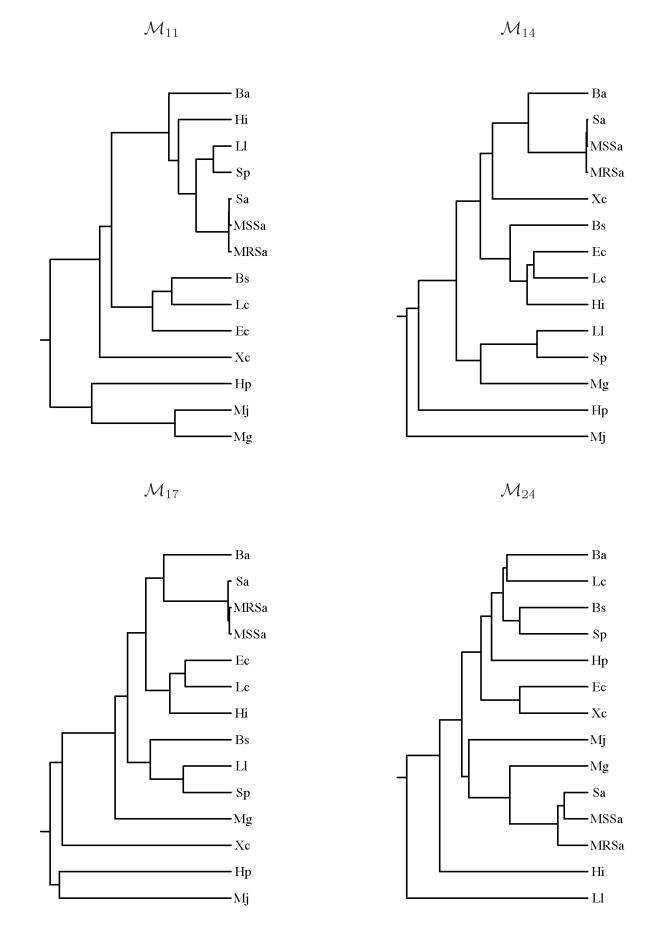
Similarity in dinucleotide compositional biases in prokaryotes:. Dendograms from dinucleotide relative abundances in sets of minimal absent words of word length 11 (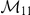), 14 (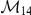), 17 (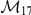) and 24 (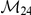) for selected prokaryotic genomes.

**Figure 3:**
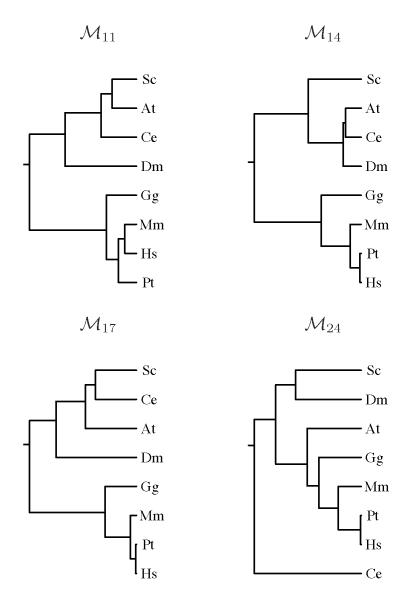
Similarity in dinucleotide compositional biases in eukaryotes. Dendograms from dinucleotide relative abundances in sets of minimal absent words of word length 11 (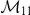), 14 (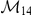), 17 (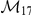) and 24 (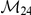) for selected eukaryotic genomes.

Figures 2 and 3 display dendograms of the similarity in dinucleotide compositional biases amongst organisms and throughout different sets of minimal absent words. Dendograms are obtained from matrices with the pairwise Euclidean distances between distinct vectors (of length 16) of dinucleotide relative abundances (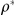 values in Table 3), using the unweighted pair group method with arithmetic averages (UPGMA, also known as average linkage method [22]). UPGMA is a simple hierarchical clustering method that, by assuming a constant rate of evolution, hence no implicit evolutionary model, outputs a rooted tree where the sum of times down a path to the leaves from any node is the same, regardless of the chosen path. Dendograms were drawn using the PHYLIP package [23]. These dendograms based on dinucleotide relative abundances provide a very useful normalization of often very differently sized sets of minimal absent words, and they are preferred to dendograms resulting from multiple sequence alignments due to current algorithmic limitations that render practically infeasible to consider such large data sets as those in sets 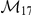.

The dendograms of similarity in dinucleotide relative abundances displayed in Figures 2 and 3 often do not recover the correct phylogenetic relationships, as dendograms based on whole genome data would, because sets of minimal absent words can have compositional biases very different from those of the genome (Table 2). Nevertheless, they are useful for exploring the hypothesis of the inheritance of minimal absent words through a common ancestor, in addition to lineage specific inheritance, as proposed by Acquisti *et al.* [4] in different sets of minimal absent words. We find that this hypothesis is not supported by our data for organisms other than vertebrates, as these represent the only clade whose phylogenetic relationships are often recovered in these dendograms.

As minimal absent words are intrinsically related to perfect repeats, they are closely dependent upon the overall repeats content in the genome, and distinct repeat classes will be associated to sets of minimal absent words of increasing word length. The small set of 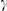-proteobacteria considered here (*E. coli*, *H. influenzae* and *X. campestris*) have, on average, higher GC content than the 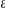-proteobacterium (*H. pylori*), the firmicutes (*B. anthracis*, *B. subtilis*, *L. casei*, *L. lactis*, *M. genitalium*, *S. aureus*, methicillin-resistant *S. aureus*, methicillin-susceptible *S. aureus* and *S. pneumoniae*), and even the euryarchaeota (*M. jannaschii*). Moreover, though the genomes of the 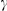-proteobacteria considered here are, on average, significantly larger than those of the other bacteria, the average percentage of generic repeats is smaller in this phylum than in the others (see [24] for statistics). The bacterium *E. coli* has one of the smallest repeat percentages of this set and its base compositional biases vary in opposition to the general trend (Table 2). This last feature is also observed in *X. campestris*, though its GC content is the highest in this set (Table 2), and its overall percentage of repeats is one of the highest.

The similarity in dinucleotide relative abundances in higher eukaryotes often recovers the phylogenetic relationships, except in set 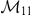, where the human is more similar to the more distantly related mouse than to the evolutionary close chimpanzee (Figure 3). Apart from the fact that these are extremely small sets in very large genomes (Table 2), we believe part of the explanation to be related to DNA transposons, which have a significant presence in both the mouse and human sets 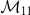 (tough larger in the latter), and which are the class of repeats that exists in more similar percentage in both genomes [25]. The separation of the worm and fruit fly from the metazoan clade may be related to the more recent origin of repeats in the worm and fruit fly than those in the remaining group (the chicken, mouse, chimpanzee and human), specially in the human genome [26].

### Conclusions

Minimal absent words, which are at a minimal distance of a single nucleotide (the left- or rightmost) from being an observed word, have been computed in the genomes of organisms from all domains of life. Here, we complement the work of Acquisti *et al.* by comparing the compositional biases of different sets of minimal absent words in the genomes of 22 organisms (one archaeota, thirteen bacteria and eight eukaryotes). We find that the mutational biases (namely, the hypermutability of CpGs) that were proposed to explain the absence of the shortest absent words in vertebrates do not explain the absence of minimal absent words, as these compositional biases are not uniform throughout different sets of minimal absent words of increasing word length. Moreover, the analysis of the similarity in dinucleotide relative abundances of different sets of minimal absent words supports the hypothesis of the inheritance of minimal absent words through a common ancestor, in addition to lineage specific inheritance, only in vertebrates.

Minimal absent words define a class of words that is closely related to perfect repeats in the genome, and not bound to protein-coding regions of the genome. Hence, we believe minimal absent words may be useful for inferring *de novo* genomic homology and potentially to uncover a plethora of new information on the evolution of genomes. Such strategy would overcome some of the major pitfalls of current genomic homology inference methods, which often fail to detect homology when there is considerable sequence divergence and mostly ignore the non-protein-coding regions of the genome [27]–[29]. This might prove to be a particularly useful methodology in genomes with high repeat content, such as the human genome, where more than half of the sequence remains ‘dark matter’, with only 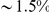 exons and 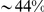 repetitive sequences presently annotated.
